# Neuroimaging Patterns and Function in Cerebral Palsy—Application of an MRI Classification

**DOI:** 10.3389/fneur.2020.617740

**Published:** 2021-02-03

**Authors:** Kate Himmelmann, Veronka Horber, Elodie Sellier, Javier De la Cruz, Antigone Papavasiliou, Ingeborg Krägeloh-Mann, The Surveillance of Cerebral Palsy in Europe (SCPE) Collaboration

**Affiliations:** ^1^Department of Pediatrics, Clinical Sciences, Sahlgrenska Academy, University of Gothenburg, Gothenburg, Sweden; ^2^Department of Paediatric Neurology, University Children's Hospital, Tübingen, Germany; ^3^Univ. Grenoble Alpes, CNRS, Grenoble INP, CHU Grenoble Alpes, TIMC-IMAG, Grenoble, France; ^4^RHEOP, Grenoble, France; ^5^Research Institute (i+12), SAMID, University Hospital “12 Octubre”, Madrid, Spain; ^6^Department of Paediatric Neurology, IASO Children's Hospital, Athens, Greece

**Keywords:** cerebral palsy, neuroimaging, MRI pattern, associated impairments, functional profile, impairment index

## Abstract

**Background:** Cerebral palsy (CP) is a disorder of movement and posture and every child with CP has a unique composition of neurological symptoms, motor severity, and associated impairments, constituting the functional profile. Although not part of the CP definition, magnetic resonance imaging (MRI) sheds light on the localization, nature, and severity of brain compromise. The MRI classification system (MRICS), developed by the Surveillance of Cerebral Palsy in Europe (SCPE), describes typical MRI patterns associated with specific timing of vulnerability in different areas of the brain. The classification has proven to be reliable and easy to use.

**Aims:** The aim of this study is to apply the MRICS on a large dataset and describe the functional profile associated with the different MRI patterns of the MRICS.

**Materials and Methods:** Data on children with CP born in 1999–2009 with a post-neonatal MRI from 20 European registers in the JRC-SCPE Central Registry was included. The CP classification and the MRICS was applied, and The Gross Motor Function Classification (GMFCS) and the Bimanual Fine Motor Function (BFMF) classification were used. The following associated impairments were documented: intellectual impairment, active epilepsy, visual impairment, and hearing impairment. An impairment index was used to characterize severity of impairment load.

**Results:** The study included 3,818 children with post-neonatal MRI. Distribution of CP type, motor, and associated impairments differed by neuroimaging patterns. Functional profiles associated with neuroimaging patterns were described, and the impairment index showed that bilateral findings were associated with a more severe outcome both regarding motor impairment and associated impairments than unilateral compromise. The results from this study, particularly the differences in functional severity regarding uni- and bilateral brain compromise, may support counseling and service planning of support of children with CP.

## Introduction

Cerebral palsy (CP) is defined as a disorder of movement and posture affecting activity. In the latest definition, accompanying impairments are acknowledged (1). Every child with CP has a unique composition of neurological symptoms, motor severity, and accompanying impairments, constituting their functional profile. National guidelines have long recommended magnetic resonance imaging (MRI) as a diagnostic step after history taking neurological examination and examination of additional impairments (2). International guidelines also view MRI as part of the work-up (3). Although not part of the CP definition, MRI sheds light on the localization, nature, and extent of brain compromise. Whether the lesions affect cerebral hemispheres uni- or bilaterally will affect the capacity for plasticity and ultimately the outcome, and insults to core structures important for network building are also of importance (4). Typical MRI patterns have been identified and associated with specific timing of vulnerability in different localizations of the brain (5, 6). Several classifications of MRI findings in CP have been proposed (7–10). One is the MRI classification system (MRICS), developed by the Surveillance of Cerebral Palsy in Europe, SCPE (11, 12). A first report of European population-based results of the MRICS was recently published (13). The quality of the MRICS data relies on training of the register partners, who, guided by the Reference and Training Manual and annual exchange and discussions, do the classification of MRI reports and scans and are instructed to classify the neuroimaging pattern which most likely is the cause of the CP. Classifications done are validated by three pediatric neurologists with specific expertise in MRI (IK-M, VH, and KH). This classification has been proved to be reliable and easy to use in this way (12).

The structure–function relationship with regard to neuroimaging findings and CP has previously been addressed by the SCPE in the Reference and Training Manual^1^ (14) and in a previous study (13), where the distribution of MRI patterns was reported by different gestational ages and CP subtypes.

Following this path, the aim of the present study was to apply the MRICS on a large population-based dataset and describe the functional profiles found, by the different MRI patterns of the MRICS. A second aim was to compare the outcome with respect to uni- and bilateral MRI findings.

## Materials and Methods

### Study Population

Data were gathered from 20 geographically defined case registers across Europe. Children with CP born between 1999 and 2009 were included if they fulfilled clinical criteria after their 4th birthday. Children whose brain damage occurred after the neonatal period (beyond the 28th day after birth) were excluded. Children with a report of MRI performed after 1 month of age were then included.

### Data Collection

The MRI classification (MRICS) of the SCPE was applied [Table 1; (12)]. The CP subtype was classified according to SCPE (11) into unilateral spastic CP (USCP), bilateral spastic CP (BSCP), dyskinetic CP, ataxic CP, or unknown. Gross motor function was classified according to the Gross Motor Function Classification (GMFCS) (15) and fine motor function according to the Bimanual Fine Motor Function (BFMF) classification (16, 17). Accompanying impairments were documented: Severe intellectual impairment was defined as an IQ below 50, tested or clinically estimated. Active epilepsy was defined as ongoing anti-epileptic medication. Severe visual impairment was defined as an acuity below 0.1 in the best eye, and severe hearing impairment was defined as hearing loss >70 dB on the better ear, before correction.

**Table 1:** The MRI classification system (12).

**A. Maldevelopments** A.1. disorders of cortical formation (proliferation and/or migration and/or organization) A.2. other maldevelopments (examples: holoprosencephaly. Dandy Walker malformation, corpus callosum agenesis, cerebellar hypoplasia)
**B. Predominant white matter injury** B.1. periventricular leucomalacia, PVL (mild/severe) B.2. sequelae of intraventricular hemorrhage (IVH) or periventricular hemorrhagic infarction (PVHI) B.3. combination of PVL and IVH sequelae
**C. Predominant gray matter injury** C.1. basal ganglia/thalamus lesions (mild/moderate/severe) C.2. cortico subcortical lesions only (watershed lesions in parasagittal distribution/multicystic encephalomalacia) not covered under C3 C.3. arterial infarctions (middle cerebral artery/other)
**D. Miscellaneous (examples**: cerebellar atrophy, cerebral atrophy, delayed myelination, ventriculomegaly not covered under B, hemorrhage not covered under B, brainstem lesions, calcifications)
**E. Normal**

*Printed with permission. IVH, intraventricular hemorrhage*.

The recently suggested impairment index (18) was applied when uni- and bilateral neuroimaging patterns were compared: *Low impairment* was defined as being able to walk (GMFCS I–II), IQ ≥70, no visual impairment, no hearing impairment, and no epilepsy. *High impairment* was defined as inability to walk (GMFCS IV-V) and/or severe intellectual impairment (IQ <50), with or without one or more of the following impairments: severe visual impairment, severe hearing impairment, and active epilepsy. *Moderate impairment* included all other levels of impairment not defined as low or high [GMFCS I–II, IQ ≥70, but with one or more of the following impairments: severe visual impairment, severe hearing impairment, active epilepsy, OR GMFCS I–II with an IQ ≥50 and <70, with or without one or more of severe visual impairment, severe hearing impairment, and/or active epilepsy OR walking with aids (GMFCS III), with an IQ ≥50 with or without one or more of the following impairments: severe visual impairment, severe hearing impairment, and active epilepsy].

### Statistical Analysis

Categorical variables were summarized as frequencies and percentages. To test for the association between imaging findings and clinical characteristics, chi-squared test or Fisher's exact test when appropriate was used. We also compared the distribution of CP types between children included in the study (with a post-neonatal MRI) and the children excluded in the study (with no report of post-neonatal MRI) using the chi-square test.

The level of statistical significance was set at 0.05.

## Results

There were 3,818 children with CP born 1999–2009 from 20 centers across Europe with an MRI performed in the post-neonatal period, while 5,415 children had no MRI report: in 1,720 (45%) children before 2 years of age (median age 12 months, interquartile range 8–16 months) and in 1,859 (49%) children at 2 years of age or later, MRI was performed. Age was unknown in 239 (6%) cases. The distribution of CP types was similar in the two groups (data not shown).

Of the 3,818 children, 1,320 (35.3%) had USCP, 1,930 (51.7%) had BSCP, 331 (8.9%) had dyskinetic CP, and 154 (4.1%) had ataxic CP, while CP type was unknown in 83 children.

The most common findings were white matter injury (49.1%) and gray matter injury (21.3%). MRI findings differed between CP types (*p* < 0.001).

**Figure 1:**
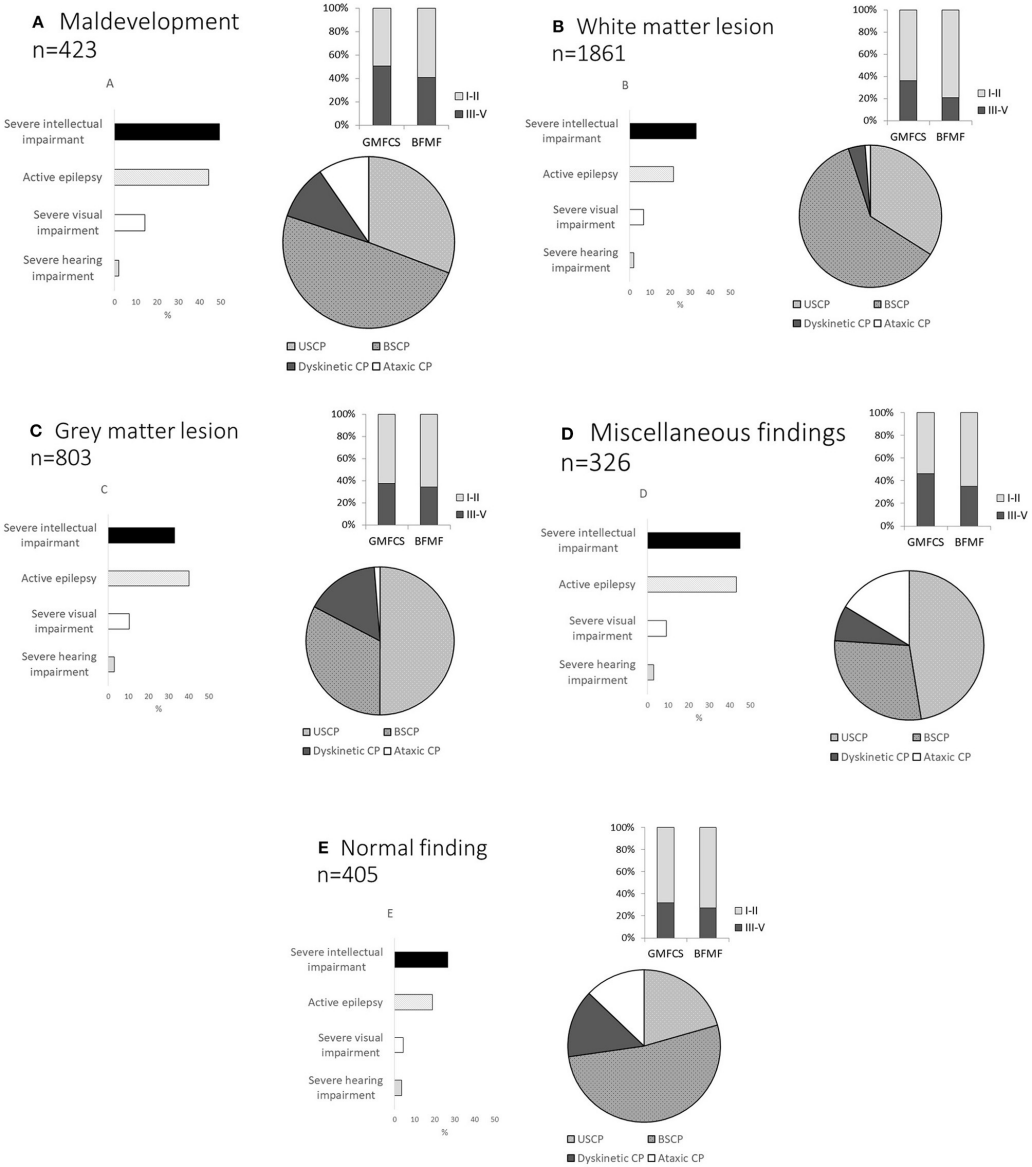
Distribution of cerebral palsy (CP) type, gross and fine motor function, active epilepsy, severe intellectual impairment, severe visual impairment, and severe hearing impairment according to MRICS (12): **(A)** maldevelopment, **(B)** white matter injury, **(C)** gray matter lesion, **(D)** miscellaneous findings, and **(E)** normal finding on MRI, Gross Motor Function Classification System (GMFCS) levels I–V, Bimanual Fine Motor Function (BFMF) levels I–V. Unilateral spastic CP (USCP), bilateral spastic CP (BSCP), dyskinetic CP, and ataxic CP.

Clinical characteristics, such as CP type, gross and fine motor function, presence of epilepsy, and severe intellectual, visual, and hearing impairment, are shown by neuroimaging pattern in Figure 1.

**Figure 2:**
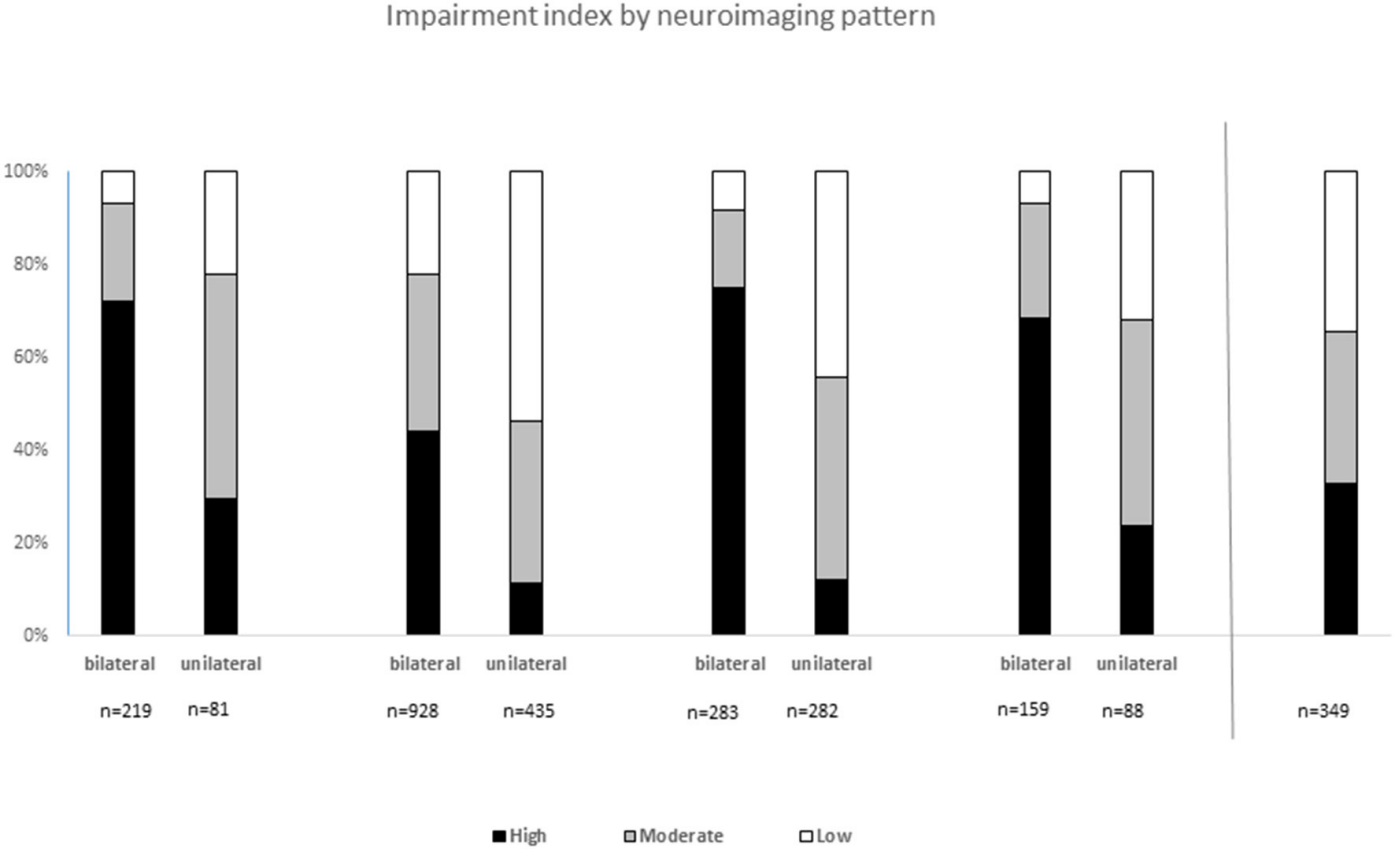
Impairment index by neuroimaging pattern and by bilateral or unilateral findings in 2,824 children with cerebral palsy: *Low impairment* was defined as being able to walk (GMFCS I-II), IQ ≥70, no visual impairment, no hearing impairment, and no epilepsy. *High impairment* was defined as inability to walk (GMFCS IV–V) and/or severe intellectual impairment (IQ <50), with or without one or more of the following impairments: severe visual impairment, severe hearing impairment, and active epilepsy. *Moderate impairment* included all other levels of impairment not defined as low or high.

Distribution of motor and accompanying impairment differed between neuroimaging patterns and whether lesions were uni- or bilateral. The impairment index was applied in 2,824 children, for whom sufficient information about impairments and MRI was registered (Figure 2).

### Neuroimaging Patterns

#### Maldevelopments

In 423 children, maldevelopment was found. This group had spastic CP in 77%, and severe intellectual impairment in 55%. Independent walkers (GMFCS I–II) comprised 47.4%, while 52.5% relied on wheelchair ambulation. Severe visual impairment was found in 14.2%, severe hearing impairment in 4.3% and active epilepsy in 46.2% (Figure 1A) Information on whether the lesion was uni- or bilateral was available in 338 children (Table 2).

**Table 2:** Comparison of subtype and associated impairments according to bilateral and unilateral lesion in children with predominant maldevelopments on brain MRI.

**Impairment**	**Localization of predominant maldevelopments**			*P*-value^*^
	**Bilateral *N* = 244 (%)**	**Unilateral *N* = 94 (%)**	**Unknown *N* = 85**	
CP type				<0.001
Spastic bilateral	149 (63.1)	16 (17.2)	35 (45.4)	
Spastic unilateral	39 (16.5)	65 (69.9)	21 (27.3)	
Dyskinetic	25 (10.6)	6 (6.4)	11 (14.3)	
Ataxic	23 (9.7)	6 (6.4)	10 (13.0)	
GMFCS III–V	155 (64.6)	17 (18.3)	42 (56.2)	<0.001
BFMF III–V	124 (59.9)	24 (30.8)	24 (46.1)	<0.001
Epilepsy				0.40
Active	114 (48.5)	37 (40.7)	37 (45.7)	
Not active	15 (6.4)	8 (8.8)	3 (3.7)	
No epilepsy	106 (45.1)	46 (50.5)	41 (50.6)	
Severe intellectual impairment	147 (66.8)	21 (25.6)	41 (51.9)	<0.001
Severe visual impairment	40 (16.4)	2 (2.1)	18 (21.2)	<0.001
Severe hearing impairment	14 (5.7)	4 (4.3)	0 (–)	0.59

* *The p-value corresponds to the comparison between bilateral and unilateral lesions. Unknown values were excluded from the comparison*.

#### White Matter Injury

Of 1,861 children, 94% had spastic CP, bilateral in almost two thirds of these cases. A majority, 63%, were independent walkers (GMFCS I–II). Only 23% (404/1,774) had active epilepsy, 6.8% had severe visual impairment, and 1% severe hearing impairment, while severe intellectual impairment was found in 24% (Figure 1B).

Information on whether the lesion was uni- or bilateral was available in 1,551 children. Children with bilateral white matter lesions mainly had BSCP, while in unilateral lesions, the majority had USCP. Severe intellectual impairment, severe visual and hearing impairments, and epilepsy were more common in children with bilateral than unilateral lesions (Table 3).

**Table 3:** Comparison of subtype and associated impairments according to bilateral and unilateral lesion in children with predominant white matter injury.

**Impairment**	**Localization of predominant white matter lesions**			*P*-value^*^
	**Bilateral *N* = 1,058 (%)**	**Unilateral *N* = 493 (%)**	**Unknown *N* = 310**	
CP type				<0.001
Spastic bilateral	850 (81.7)	86 (17.6)	182 (59.7)	
Spastic unilateral	129 (12.4)	389 (79.5)	106 (34.7)	
Dyskinetic	48 (4.6)	8 (1.6)	14 (4.6)	
Ataxic	13 (1.2)	6 (1.2)	3 (1.0)	
GMFCS III–V	510 (49.2)	48 (9.9)	117 (38.9)	<0.001
BFMF III–V	322 (38.2)	41 (10.6)	28 (25.9)	<0.001
Epilepsy				<0.001
Active	256 (25.4)	84 (17.7)	64 (21.9)	
Not active	74 (7.3)	23 (4.8)	22 (7.5)	
No epilepsy	678 (67.3)	367 (77.4)	206 (70.5)	
Severe intellectual impairment	299 (32.0)	45 (10.3)	60 (20.6)	<0.001
Severe visual impairment	86 (8.1)	15 (3.0)	26 (8.4)	<0.001
Severe hearing impairment	29 (2.7)	4 (0.8)	2 (0.6)	0.01

* *The P-value corresponds to the comparison between bilateral and unilateral lesions. Unknown values were excluded from the comparison*.

#### Gray Matter Injury

In this group of 803 children, bilateral lesions were found in 320, and unilateral in 322. The distribution as to uni- or bilateral was unknown in 150 cases (Figure 1C).

Uni- and bilateral gray matter lesions differed in outcome with a higher severity in terms of motor impairment and associated impairments for children with bilateral lesions (Table 4).

**Table 4:** Comparison of subtype and associated impairments according to bilateral and unilateral lesion in children with predominant gray matter injury.

**Impairment**	**Localization of predominant gray matter lesions**			*P*-value^*^
	**Bilateral *N* = 320 (%)**	**Unilateral *N* = 322 (%)**	**Unknown *N* = 161 (%)**	
CP type				<0.001
Spastic bilateral	178 (56.7)	30 (9.3)	51 (32.1)	
Spastic unilateral	32 (10.2)	284 (88.2)	82 (51.6)	
Dyskinetic	101 (32.2)	4 (1.2)	23 (14.5)	
Ataxic	3 (1.0)	4 (1.2)	3 (1.9)	
GMFCS III–V	232 (73.9)	13 (4.1)	56 (35.2)	<0.001
BFMF III–V	202 (74.3)	43 (16.6)	26 (54.2)	<0.001
Epilepsy				<0.001
Active	158 (50.3)	92 (29.6)	73 (49.0)	
Not active	30 (9.5)	30 (9.6)	11 (7.0)	
No epilepsy	126 (40.1)	189 (60.8)	69 (43.9)	
Severe intellectual impairment	179 (62.8)	30 (10.6)	56 (36.6)	<0.001
Severe visual impairment	48 (15.0)	7 (2.2)	29 (18.0)	<0.001
Severe hearing impairment	21 (6.6)	2 (0.7)	1 (0.6)	<0.001

* *The P-value corresponds to the comparison between bilateral and unilateral lesions. Unknown values were excluded from the comparison*.

Children with bilateral gray matter lesions had the highest proportion of dyskinetic CP, 32% (101/320).

In children with unilateral gray matter lesions, spastic CP dominated (97.5%) and this was mainly unilateral.

#### Miscellaneous Findings

Miscellaneous findings on MRI were found in 326 children, 74.5% had spastic CP, 10.7% dyskinetic, and 10% ataxic CP. GMFCS levels were at III–V in 42.5%. Active epilepsy and severe intellectual impairment were common, found in 40 and 49.7% respectively (Figure 1D). Information on whether the lesion was uni- or bilateral was available in 270 children (Table 5).

**Table 5:** Comparison of subtype and associated impairments according to bilateral and unilateral lesion in children with miscellaneous findings on brain MRI.

**Impairment**	**Localization of miscellaneous findings**			*P*-value^*^
	**Bilateral *N* = 175 (%)**	**Unilateral *N* = 95 (%)**	**Unknown *N* = 56**	
CP type				<0.001
Spastic bilateral	104 (61.5)	23 (24.5)	23 (47.9)	
Spastic unilateral	17 (10.1)	66 (70.2)	10 (20.8)	
Dyskinetic	27 (16.0)	4 (4.3)	4 (8.3)	
Ataxic	21 (12.4)	1 (1.1)	10 (20.8)	
GMFCS III–V	110 (64.7)	15 (16.0)	26 (49.1)	<0.001
BFMF III–V	78 (52.7)	21 (25.0)	16 (42.1)	<0.001
Epilepsy				0.003
Active	79 (48.2)	30 (31.9)	32 (29.6)	
Not active	20 (12.2)	6 (6.4)	6 (11.1)	
No epilepsy	65 (39.6)	58 (61.7)	32 (59.3)	
Severe intellectual impairment	101 (63.5)	21 (23.6)	25 (52.1)	<0.001
Severe visual impairment	24 (13.7)	0 (–)	6 (10.7)	<0.001
Severe hearing impairment	9 (5.1)	3 (3.2)	1 (1.8)	0.55

* *The p-value corresponds to the comparison between bilateral and unilateral lesions. Unknown values were excluded from the comparison*.

#### Normal Finding

Children with normal findings on post-neonatal MRI had a spastic CP in 70% (283/405), were able to walk independently (GMFCS I–II) in 68%, had active epilepsy in 19% (74/405), and had severe intellectual impairment in 26.5% (94/405) (Figure 1E).

In summary, in children with maldevelopment, bilateral gray matter lesions, and miscellaneous findings on MRI, a more severe pattern of impairments was frequent, both regarding motor and associated impairments. In contrast, children with white matter injury and normal finding on MRI more often had a milder phenotype.

Children with bilateral white and gray matter lesions more frequently had a severe outcome than those with unilateral lesions.

The impairment index was applied in 2,824 children with sufficient information and revealed significant differences (*p* < 0.001, respectively) between uni- and bilateral MRI findings regardless of MRICS classification A through D, with a larger proportion of high impairment index in bilateral findings (Figure 2). Children with white matter lesions (B) overall had the least severe impairment load.

### CP Types

Spastic CP was the most frequent in all MRI patterns. Less common CP types, such as dyskinetic CP and ataxic CP, were unevenly distributed. Most cases of dyskinetic CP were found in the group with bilateral gray matter injury.

Bilateral spastic CP was the most common CP subtype. Of the 1,118 children, severe motor and associated impairments were most frequent in those with maldevelopments or gray matter lesions (Table 6).

**Table 6:** Comparison of associated impairments according to main lesion in children with bilateral spastic CP.

**Impairment**	**MRI finding**					***P*-value**
	**A**	**B**	**C**	**D**	**E**	
	***N* = 200**	***N* = 1,118**	***N* = 259**	***N* = 150**	***N* = 203**	
GMFCS III–V	157 (80.1)	594 (54.0)	189 (74.4)	96 (66.7)	73 (36.9)	<0.001
BFMF III–V	118 (72.8)	319 (39.2)	137 (74.5)	69 (59.0)	31 (23.8)	<0.001
Epilepsy						<0.001
Active	109 (56.2)	282 (26.4)	143 (56.3)	65 (46.1)	35 (18.0)	
Not active	14 (7.2)	76 (7.1)	25 (9.8)	17 (12.1)	11 (5.7)	
No epilepsy	571 (36.6)	708 (66.4)	86 (33.9)	59 (41.8)	148 (76.3)	
Severe intellectual impairment	135 (73.0)	312 (31.3)	166 (70.0)	86 (64.2)	53 (29.3)	<0.001
Severe visual impairment	45 (22.5)	94 (8.4)	59 (22.8)	17 (11.3)	11 (5.4)	<0.001
Severe hearing impairment	10 (5.0)	24 (2.1)	12 (4.6)	7 (4.7)	5 (2.5)	0.05

In unilateral spastic CP, two different neuroimaging patterns dominated: white matter lesion (B) and gray matter lesion (middle cerebral artery infarction) (C), emanating from different timing of compromise. The two groups had a similar profile of impairment index but differed with regard to prevalence of accompanying impairments (Table 7). Children with unilateral spastic CP and gray matter lesion or maldevelopment had a higher proportion of severe intellectual impairment and epilepsy than those with white matter lesions.

**Table 7:** Comparison of associated impairments according to main lesion in children with unilateral spastic CP.

**Impairment**	**MRI finding**					***P*-value**
	**A**	**B**	**C**	**D**	**E**	
	***N* = 125**	***N* = 624**	***N* = 398**	***N* = 93**	***N* = 80**	
GMFCS III–V	8 (6.4)	15 (2.4)	7 (1.8)	7 (7.5)	0 (–)	0.002
BFMF III–V	16 (15.4)	30 (6.9)	43 (15.6)	9 (11.0)	2 (3.7)	0.001
Epilepsy						<0.001
Active	43 (35.2)	90 (14.9)	126 (32.6)	28 (30.1)	7 (8.9)	
Not active	10 (8.2)	27 (4.5)	32 (8.3)	7 (7.5)	6 (7.6)	
No epilepsy	69 (56.6)	485 (80.6)	228 (59.1)	58 (62.4)	66 (83.5)	
Severe intellectual impairment	19 (17.8)	38 (6.7)	40 (11.1)	15 (16.8)	3 (4.3)	<0.001
Severe visual impairment	3 (2.4)	18 (2.9)	9 (2.3)	2 (2.1)	0 (–)	0.62
Severe hearing impairment	6 (4.8)	7 (1.1)	3 (0.7)	1 (1.1)	1 (1.2)	0.01

## Discussion

Neuroimaging is an important tool in disclosing clues to the background of CP. More than 80% of brain imaging in CP is abnormal in most studies. To achieve a common language regarding neuroimaging findings, the SCPE has suggested a classification describing the different patterns of abnormality, based on the timing of insult to the brain (12). This gives a possibility to investigate structure–function relationships and may help in predicting future impairments and increase the possibilities of prevention and treatment. Earlier studies have suggested a relationship between lesions occurring late in gestation and impaired speech and communication (19), and outcome of gross and fine motor function are also found to be related to timing of the lesion (4, 20, 21). Extent and topography of the lesions also play a role. Whether lesions affect cerebral hemispheres uni- or bilaterally is crucial for functional outcome. Following early brain lesions that are unilateral, the brain can refer to homotopic areas of the healthy hemisphere. This potential for reorganization is unique to the young brain. With respect to motor function, ipsilateral motor tracts can be recruited, but with relevant functionality only in earlier brain lesions, e.g., before the end of the third trimester of pregnancy or equivalent preterm age (22). Language can be reorganized to the right after early left hemispheric lesions up to early childhood age, as the representation of the language network is initially bilateral, whereas the young brain is more sensitive and vulnerable to lesions when these are bilateral and interfere with early network building (4, 23).

This study aimed to describe functional profiles by the neuroimaging patterns of the MRICS, using the large population-based database of the SCPE (11). A previous report from this database stated that the neuroimaging findings were mainly lesional, while maldevelopment, miscellaneous, and normal findings constituted smaller groups (13) and studies from national registers find similar results (24, 25). The present study shows that the impairment load in terms of occurrence of severe intellectual, visual, and hearing impairment and epilepsy, as well as in the distribution of gross and fine motor impairment, differs between neuroimaging patterns. Moreover, the severity of motor function and occurrence of associated impairments differs between children with unilateral and bilateral lesions, the latter associated with a more severe phenotype, in all neuroimaging patterns. As discussed above, homotopic areas in an unaffected hemisphere offer the possibility to reorganize and compensate for function, whereas vulnerability of the young brain to bilateral lesions is probably higher than in later age (4). This finding was supported when applying the impairment index (18), showing a significant difference in impairment load between uni- and bilateral maldevelopments or lesions, regardless of timing of event during gestation. It is noteworthy that unilateral white matter injuries (representing mostly periventricular hemorrhagic infarctions of preterm children, former IVH grade IV) present with the least impairment load, even less than unilateral gray matter injuries (e.g., arterial infarctions). We consider this an important aspect in the early counseling of the parents of a preterm child.

An increasing number of associated impairments have been recognized in children with CP. Recent contributions are neuropsychiatric disorders, reported to be associated with male sex, epilepsy, and intellectual disability in European children with CP (26). Other studies have pointed toward an association with white matter injuries (27, 28). Early insults to the brain may predict both attention and executive functioning (29, 30). Disruption of structural brain connectivity has been found, not only in the sensorimotor system but also in posterior brain regions, associated with intellectual impairment in individuals with dyskinetic CP (31). Thus, many factors affect the occurrence of impairment in children with early insult to the brain, and much is still unknown.

Limitations to this study must be recognized. The data came from the largest population-based database on children with CP worldwide. However, not all children had been subject to MRI. Also, there were missing data of whether the neuroimaging findings were uni- or bilateral. Information about intellectual impairment was in some cases based on estimation, often due to difficulties performing formal tests in severe cases.

However, the existing data provide a large basis to clarify structure–function relationships in the heterogeneous population of children with CP, illustrating that the proportion of severe impairments is clearly larger in children with bilateral lesions, consistent with previous findings (4). Moreover, the classification of MRI reports and scans was done and validated in a structured and uniform way, strengthening data quality.

Further insights in the association between early brain compromise and clinical outcome in CP may be gained using a harmonized classification of neuroimaging findings such as the MRICS as a basis for research. As suggested earlier (12), additional information with regard to exact topography and extent of the brain lesions must be taken into account (32, 33). Such insights must be combined with additional knowledge about the child, for example regarding cognitive functioning (34).

We conclude that the distribution of motor and associated impairments differed between neuroimaging patterns in children with CP. A consistent finding was that bilateral lesions or maldevelopment more often gave a severe phenotype. This information may support individual counseling and planning of support of the child with CP.

## Data Availability Statement

The datasets presented in this article are not readily available because the dataset is derived from the Central Database of JRC-SCPE, where requests should be directed. Requests to access the datasets should be directed to JRC-SCPE@ec.europa.eu.

## Author Contributions

KH drafted and revised the manuscript. VH, ES, JD, AP, and IK-M contributed to and critically revised several versions of the manuscript. The SCPE Collaboration contributed to and critically revised a final manuscript together with KH, VH, ES, JD, AP, and IK-M. All authors contributed to the article and approved the submitted version.

## SCPE Collaboration (Registry Leaders)

Elodie Sellier (RHEOP, Grenoble, France), Catherine Arnaud (RHE31, Toulouse, France), Oliver Perra (NICPR, Belfast, UK), Kate Himmelmann (Gothenburg, Sweden), Owen Hensey (Dublin, Ireland), Karen Horridge (Sunderland, UK), Mary Jane Platt (Norwich, UK), Gija Rackauskaite (Copenhagen, Denmark), Marco Marcelli (Viterbo, Italy), Guro L. Andersen (Tonsberg, Norway), Javier De la Cruz (Madrid, Spain), David Neubauer (Ljubljana, Slovenia), Daniel Virella (Lisbon, Portugal), Antigone Papavasiliou (Athens, Greece), Andra Greitane (Riga, Latvia), Katalin Hollody (Pecs, Hungary), Solveig Sigurdardottir (Kopavogur, Iceland), Fiona Zeiner (Innsbruck, Austria), Vlatka Mejaski-Bosnjak (Zagreb, Croatia), Els Ortibus (Leuven, Belgium), Ingeborg Krägeloh-Mann (Tübingen, Germany), Stephen Attard (Malta), and Ecaterina Bufteac (Chisinau, Moldova).

## Conflict of Interest

The authors declare that the research was conducted in the absence of any commercial or financial relationships that could be construed as a potential conflict of interest.

## Footnotes

^1^https://eu-rd-platform.jrc.ec.europa.eu/scpe/reference-and-trainingmanual_en (accessed October 14, 2020).

## Abbreviations

BFMF: Bimanual Fine Motor Function
BSCP: bilateral spastic cerebral palsy
CP: cerebral palsy
GMFCS: Gross Motor Function Classification System
MRI: magnetic resonance imaging
USCP: unilateral spastic cerebral palsy.
